# Site-specific relapse pattern of the triple negative tumors in Chinese breast cancer patients

**DOI:** 10.1186/1471-2407-9-342

**Published:** 2009-09-24

**Authors:** Yanping Lin, Wenjin Yin, Tingting Yan, Liheng Zhou, Genhong Di, Jiong Wu, Zhenzhou Shen, Zhimin Shao, Jinsong Lu

**Affiliations:** 1 Department of General Surgery, Shanghai Sixth People's Hospital, Shanghai Jiaotong University, Shanghai 200233, PR China; 2 Department of Breast Surgery, Cancer Hospital/Cancer Institute, Fudan University, Shanghai 200032, PR China; 3 Department of Oncology, Shanghai Medical College, Fudan University, Shanghai 200032, PR China

## Abstract

**Background:**

It has been reported that triple negative phenotype is characterized by aggressive clinical history in Western breast cancer patients, however its pattern of metastatic spread had never been reported in the Chinese population. Considering racial disparities, we sought to analyze the spread pattern for different sites of first recurrence in Chinese triple negative breast cancers.

**Methods:**

A retrospective study of 1662 patients was carried out from a large database of breast cancer patients undergoing surgery between January 1, 2000 and March 31, 2004 at the Cancer Hospital, Fudan University, Shanghai, China. Survival curves were generated using the Kaplan-Meier method and annual relapse hazards were estimated by the hazard function.

**Results:**

We found a statistically significant difference in relapse-free survival (RFS) for locoregional and visceral recurrence (*P *= 0.007 and *P *= 0.025, respectively) among the triple negative, ERBB2+ and HR+/ERBB2- subgroups in univariate analysis. In the multivariate Cox proportional hazards regression analysis, RFS for either locoregional or visceral relapse in the triple negative category was inferior to that in HR+/ERBB2- patients (*P *= 0.027 and *P *= 0.005, respectively), but comparable to that in ERBB2+ women (both *P *>*0.05*). Furthermore, the early relapse peak appeared later in the triple negative group than that in the ERBB2+ counterpart for both locoregional and visceral relapse. On the other hand, when compared with triple negative breast cancers, a significantly lower risk of developing bone relapse was discerned for ERBB2+ women (*P *= 0.048; HR = 0.384, 95% CI 0.148-0.991), with the borderline significance for HR+/ERBB2- breast cancers (*P *= 0.058; HR = 0.479, 95% CI 0.224-1.025). In terms of bone metastasis, the hazard rate remained higher for the triple negative category than that for the ERBB2+ subtype.

**Conclusion:**

Based on the site-specific spread pattern in different subgroups, the triple negative category of breast cancers in the Chinese population exhibits a different pattern of relapse, which indicates that different organotropism may be due to the different intrinsic subtypes. A better knowledge of the triple negative category is warranted for efficacious systemic regimens to decrease and/or delay the relapse hazard.

## Background

Recent advances in genomic techniques have led to the classification of breast cancer into five distinct subtypes: the luminal A, luminal B, ERBB2+, normal breast-like and basal-like subtype [1]. The fifth group is roughly synonymous with triple negative breast cancer, featuring absent expression of estrogen, progesterone and ERBB2 receptors. Although systemic therapy, together with targeted treatment, has revealed a considerable impact on the improvement of prognosis, there is to date no recommended regimen for triple negative women due to the scarcity of data as well as the dearth of target.

The triple negative category generally makes up 10-15% of breast cancers [2]. However, the prevalence varies widely by race. This subgroup occurred at a higher incidence (39%) in pre-menopausal African American women [3], whereas there was a rather lower frequency (18%) in Chinese patients [4], much similar to the Japanese series (8-14%) [5,6]. Manifold data demonstrated the detrimental effect of triple negative phenotype on survival in Western populations [3,7-13], as opposed to more favorable prognosis in Chinese as well as Japanese counterparts [4,5]. Despite racial preference, these results provide support for the increasing recognition that breast cancer is a heterogeneous disease categorized as different subgroups with a wide spectrum of clinical, pathological and molecular features [8]. Therefore, it hints at a demand for further investigation on the intrinsic characteristics of triple negative tumors in different populations including Chinese patients.

Nowadays, there is an increasing interest in hazard function, which highlights changes of the event probability over time. When it comes to the triple negative category, its unique time distribution of recurrence risk, as distinguished from that for the other subtypes, has been reported in both Western series and our previous study [4,10]. Nevertheless, such information is still not available with regard to different sites of first recurrence. Current data indicate that the triple negative group is more prone to visceral metastases, local relapse and cerebral metastases rather than bone metastases as compared to women with non-triple negative tumors [13-15]. Unfortunately, prior studies were almost confined to Western populations and almost conducted by way of dichotomization. And most dichotomized cases according to hormone receptor status or as triple negative/non-triple negative, which obscures the superiority of ERBB2 status to hormone receptor (HR) status as a prognosticator in breast cancer [16]. Considering the discrepancies in races and subgroups, it remains unclear whether the site-specific relapse pattern is similar or not for Chinese triple negative patients, which serves as a reminder of the need for exploration to promote a better understanding of organotropic nature between various races and subgroups.

In view of the above points, a retrospective analysis was carried out to further elucidate the spread pattern for different sites of first recurrence in Chinese triple negative breast cancer patients as compared to ERBB2+ and HR+/ERBB2- peers. In this way, we sought to get a clear picture of the predisposition to organ metastases for Chinese triple negative breast cancer patients, thereby, shedding more light on the underlying distinction in biological behavior between different subgroups.

## Methods

### Patients

A total of 1662 patients were selected retrospectively from a large database of patients who underwent surgery between January 1, 2000 and March 31, 2004 at the Cancer Hospital, Fudan University, Shanghai, China.

Before surgery, the following evaluations were mandatory for each patient as complete physical examination, chest radioscopy, bilateral mammography, ECG, ultrasonography of breasts, axillary fossa, cervical parts, abdomen, and pelvis, complete blood count, and routine biochemical tests to make an exact staging. Thereafter, each patient received lumpectomy or mastectomy followed by adjuvant therapy according to the guidelines or recommendations used at the time of surgery.

Eligibility criteria for this analysis, similar to other relevant reports [17,18], included female gender, an initial diagnosis of unilateral primary breast cancer without distant metastases, at least 2 months of follow-up information for disease recurrence, and complete data on the following: age, tumor size, number of involved axillary lymph nodes, status of estrogen receptor (ER), progesterone receptor (PR) as well as ERBB2. All eligible patients in the database were included. All data were entered into a computerized database and verified to minimize errors in data entry.

Follow-up information regarding tumor recurrence and survival status was accomplished through patients' clinic visits with records kept in the computerized database of the outpatient department, personal contact with the patient as well as the assistance of Shanghai Center for Disease Control and Prevention (CDC). Thereinto, personal contact with the patient referred to routine correspondence or telephone visits, which were carried out at the Cancer Hospital, Fudan University every 3 months during the first two years, every 6 months during the next two years and once a year thereafter. As this was a retrospective study without any medical intervention, the ethical approval was not required.

### Immunohistochemistry

Immunohistochemical staining was carried out as a standard operating procedure in the pathology department of Cancer Hospital, Fudan University, Shanghai, China. All primary monoclonal antibodies for estrogen receptor (ER), progesterone receptor (PR) and ERBB2 were from Dako. The staining results were assessed by at least two pathologists, using a semiquantitative scoring system, where integrated the proportion score and the intensity score. The proportion score, indicating the percentage of tumor cells stained, was interpreted such that a score of 0 required no staining seen, 1 required ≤25% of cells positive, 2 required 25-50% of cells stained, 3 required 50-75% of positive cells and 4 required >75% of staining cells. As to the intensity score, a negative result was defined as a score of 0, weakly positive as 1, moderately positive as 2, and strongly positive as 3. The final score was calculated as the product of the proportion score and the intensity score. Thereby, staining results ranged from score 0 to 12. The scoring system for ER and PR was defined as negative for score 0 and positive for scores of 1~12 with the nucleic staining of carcinoma cells, whereas ERBB2 was defined as negative for scores of 0~8 (namely, 0, 1+ and 2+ in the DAKO scoring system) and positive for strong membranous staining with scores of 9~12 (namely DAKO score 3+).

### Statistical analysis

Clinicopathological parameters were compared between different subgroups using one-way analysis of variance (ANOVA) test for continuous variables, chi-square test for unordered categorical variables and nonparametric Kruskal-Wallis rank test for ordinal categorical variables.

Site-specific relapse-free survival (RFS) was defined as the time from surgery to the earliest occurrence of an event, including locoregional, visceral and bone relapse. Locoregional failure was defined as a first relapse on the chest wall or in the ipsilateral breast, the ipsilateral axilla, the ipsilateral supraclavicular or infraclavicular fossa, or the ipsilateral internal mammary region [19], which was required to be identified by biopsy or fine needle aspiration. Visceral metastasis was established if there was any radiological evidence of metastases to viscera (including lung, liver and brain). Bone relapse was defined as metastases to bone without visceral metastasis. Any suspicious lesions on bone scan should be confirmed by further X-ray or CT/MRI examination. Those without any evidence of event were censored at the last date they were known to be alive.

Survival analyses were estimated by the Kaplan-Meier method and were compared using the log-rank test. Multivariate Cox proportional hazards regression analyses were applied to modeling the relationship between subgroup and RFS, adjusted for age (≤50 *vs. *>50), tumor size (≤2 cm *vs. *>2 cm), number of axillary lymph nodes (ALNs) involved (0, 1-3, ≥4), histological grade (I/II *vs. *III) and systemic treatment (yes *vs. *no). Hazard ratios (HRs) were presented with their 95% confidence intervals (CIs). For graphical display of RFS, annual hazard rates were estimated using a Kernel method of smoothing. All statistical tests were two sided and *P *< 0.05 was considered to be statistically significant. All statistical analyses were performed with Stata statistical software package (release 10.0; Stata Corporation, College Station, Texas, USA).

## Results

### General characteristics

The mean age at diagnosis was 52.7 years old (range 24-90). Median follow-up was 3.15 years, ranging from 2 months to 7.8 years. According to different combinations of HR and ERBB2 status, 1662 patients were classified into the three subgroups as follows: triple negative (19.31%), ERBB2+ (26.72%) and HR+/ERBB2- (53.97%, HR+ referred to ER+ or PR+). When compared with triple negative and ERBB2+ tumors, less relapse events were observed in HR+/ERBB2- patients (15.89% and 14.64% *vs. *8.81%, *P *< 0.001; Table 1).

**Table 1:** Summary of subgroup characteristics in 1662 patients

Variables (%)	Triple negative	ERBB2+	HR+/ERBB2-	*P *value
		
	n = 321	n = 444	n = 897	
Mean age at diagnosis (year, ± SD)	52.02 ± 10.49	52.12 ± 9.99	53.16 ± 11.56	0.416
Tumor size				
≤ 2 cm	139 (43.30)	148 (33.33)	439 (48.94)	< 0.001
>2 cm	182 (56.70)	296 (66.67)	458 (51.06)	
Number of ALNs involved				
0	190 (59.19)	233 (52.48)	538 (59.98)	
1-3	72 (22.43)	107 (24.10)	214 (23.86)	0.020
≥ 4	59 (18.38)	104 (23.42)	145 (16.16)	
Histological grade				
I/II	151(61.56)	237 (74.29)	499 (81.80)	0.002
III	60 (28.44)	82 (25.71)	111 (18.20)	
Not known	110	126	287	
Systemic treatment				
Yes	302 (94.08)	426 (95.95)	852 (94.98)	0.494
No	19 (5.92)	18 (4.05)	45 (5.02)	
All relapse				
Yes	51 (15.79)	65 (14.64)	79 (8.81)	< 0.001
No	270 (84.11)	379 (85.36)	818 (91.19)	
Locoregional relapse				
Yes	25 (7.79)	35 (7.79)	35 (3.90)	0.003
No	296 (92.21)	409 (92.12)	862 (96.10)	
Mean TTE (years)	2.19	2.03	2.02	------
Visceral relapse				
Yes	15 (4.67)	25 (5.63)	23 (2.56)	0.014
No	306 (95.33)	419 (94.37)	874 (97.44)	
Mean TTE (years)	1.64	2.02	2.49	------
Bone relapse				
Yes	12 (3.74)	7 (1.58)	16 (1.78)	0.074
No	309 (96.26)	437 (98.42)	881 (98.22)	
Mean TTE (years)	3.04	2.24	2.57	------

Abbreviations: HR = hormone receptor; SD = standard deviation; ALNs = axillary lymph nodes; TTE = time to event

### Locoregional RFS and relapse hazard

**Figure 1:**
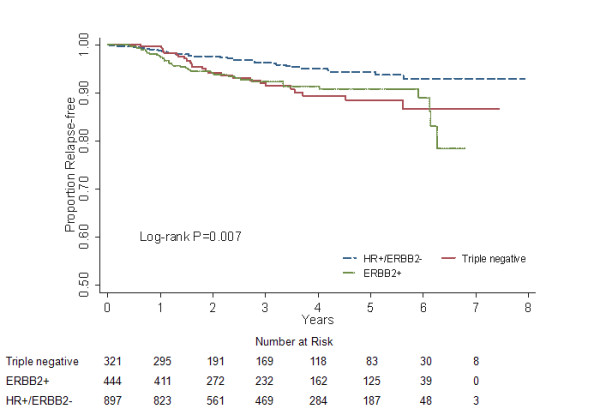
**Locoregional relapse-free survival for different subgroups in 1662 breast cancer patients**.

**Figure 2:**
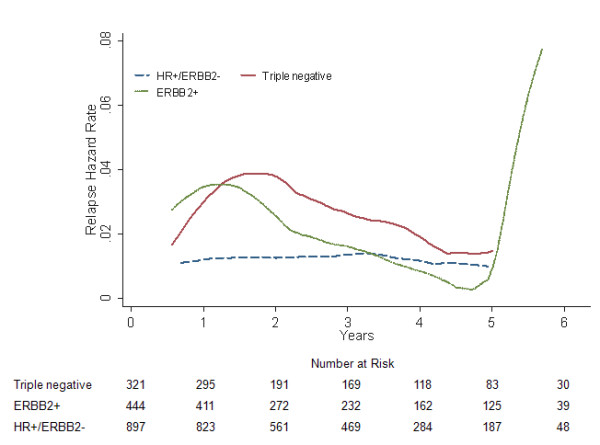
**Annual locoregional relapse hazard rates for different subgroups in 1662 breast cancer patients**.

We found a statistically significant difference in locoregional RFS between the three subgroups (log-rank *P *= 0.007, Fig. 1; adjusted *P *< 0.05, data not shown). Compared with the triple negative patients, locoregional RFS was significantly better for HR+/ERBB2- counterparts (*P *= 0.027; HR = 0.521, 95% CI 0.293-0.927; Table 2), but nearly comparable to ERBB2+ tumors (*P *= 0.221; HR = 0.686, 95% CI 0.375-1.254; Table 2). Besides, the survival curves of locoregional failure for triple negative and ERBB2+ patients were virtually superimposable. Somewhat differently in the hazard curves, the former showed an early major surge reaching the maximum at approximately 2 years after surgery, while the corresponding peak for the latter was at 1.5 years (Fig. 2).

**Table 2:** Cox proportional hazards regression analysis of RFS for locoregional, bone and visceral relapse in 1662 breast cancer patients

Subgroup*	Univariate Cox model, HRs (95% CI)	*P *value	Multivariate Cox model, HRs (95% CI)	*P *value
Locoregional RFS				
ERBB2+	1.018(0.609-1.700)	0.947	0.686(0.375-1.254)	0.221
HR+/ERBB2-	0.522(0.312-0.873)	0.013	0.521(0.293-0.927)	0.027
Visceral RFS				
ERBB2+	1.203(0.634-2.281)	0.572	0.952(0.479-1.893)	0.889
HR+/ERBB2-	0.568(0.296-1.090)	0.089	0.339(0.158-0.724)	0.005
Bone RFS				
ERBB2+	0.468(0.183-1.198)	0.113	0.384(0.148-0.991)	0.048
HR+/ERBB2-	0.553(0.259-1.178)	0.124	0.479(0.224-1.025)	0.058

*Triple negative category was used as the reference group.

Abbreviations: RFS = relapse-free survival; HRs = hazard ratios; CI = confidence interval; HR = hormone receptor

### Visceral RFS and relapse hazard

**Figure 3:**
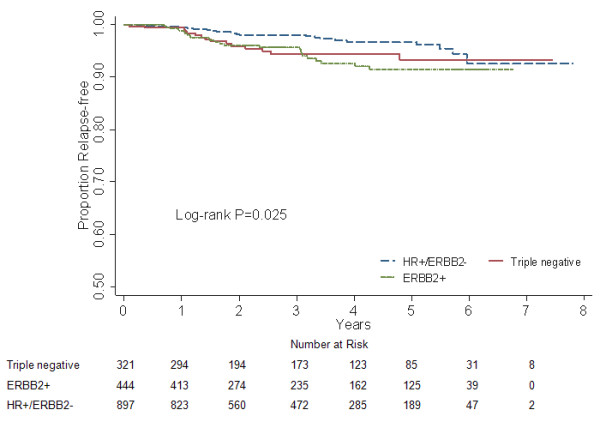
**Visceral relapse-free survival for different subgroups in 1662 breast cancer patients**.

**Figure 4:**
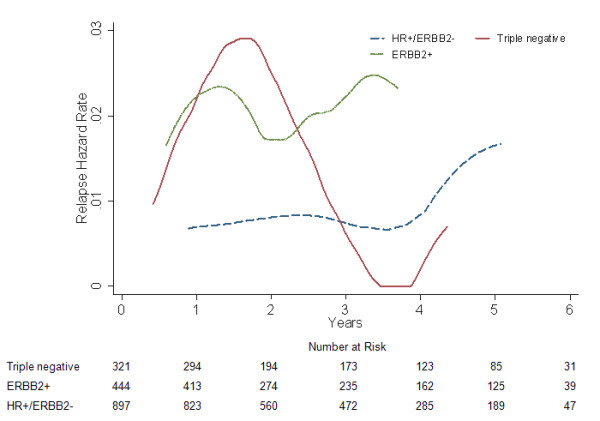
**Annual visceral relapse hazard rate for different subgroups in 1662 breast cancer patients**.

A statistically significant difference was yielded in visceral RFS among the three subgroups (log-rank *P *= 0.025, Fig. 3; adjusted *P *< 0.05, data not shown). The multivariate Cox regression analysis demonstrated that triple negative patients, similar to ERBB2+ counterparts (*P *= 0.889; HR = 0.952, 95% CI 0.479-1.893; Table 2), tended to have more visceral relapses than HR+/ERBB2- women (*P *= 0.005; HR = 0.339, 95% CI 0.158-0.724; Table 2) across all time periods. Furthermore, the early relapse peak appeared to occur later in triple negative phenotype than that in ERBB2+ subgroup, as opposed to a relatively flat hazard rate until 4 years for HR+/ERBB2- category (Fig. 4).

### Bone RFS and relapse hazard

**Figure 5:**
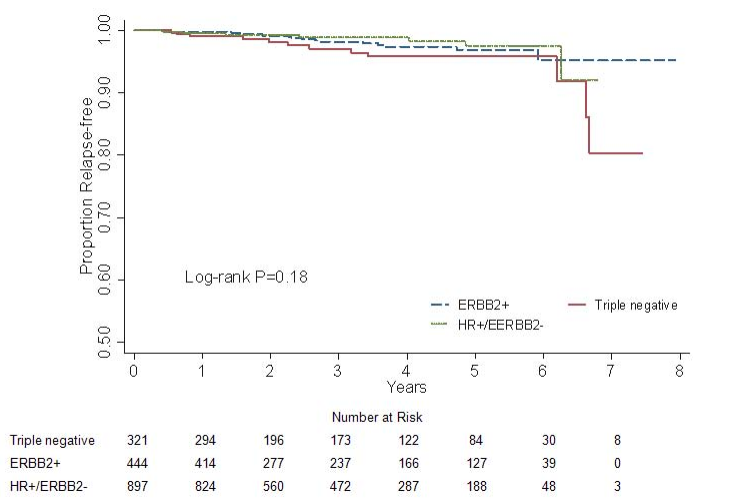
**Bone relapse-free survival for different subgroups in 1662 breast cancer patients**.

**Figure 6:**
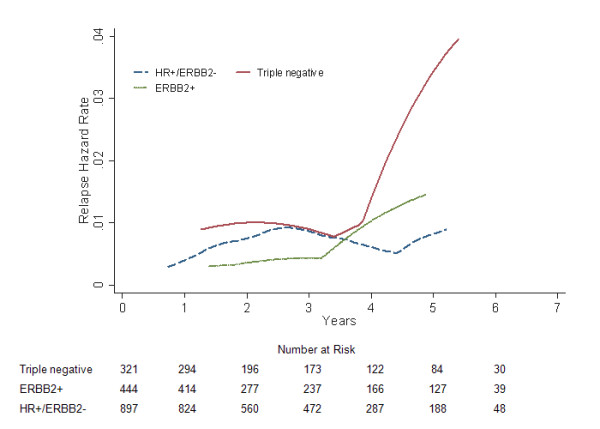
**Annual bone relapse hazard rates for different subgroups in 1662 breast cancer patients**.

There was no difference in RFS among the three groups in the univariate survival analysis (log-rank *P *= 0.180; Fig 5). Nevertheless, when compared to triple negative patients, a significantly lower risk of developing bone relapse was discerned in the multivariate Cox regression analysis for ERBB2+ women (*P *= 0.048; HR = 0.384, 95% CI 0.148-0.991), with the borderline significance for HR+/ERBB2- breast cancers (*P *= 0.058; HR = 0.479, 95% CI 0.224-1.025; Table 2). This finding was confirmed by the further analysis of hazard function (Fig. 6).

## Discussion

This study is, to the best of our knowledge, the first as well as the largest retrospective analysis on site-specific relapse pattern for triple negative tumors in Chinese breast cancer patients. As to locoregional failure, we demonstrated that RFS for the triple negative category was inferior to that for HR+/ERBB2- patients, but comparable to that for ERBB2+ women. Up to now, several studies have been reported with conflicting results. According to Rodriguez-Pinilla's reports, a higher percentage of local recurrence arose in triple negative tumors [13], which was somewhat congruent with our analysis. In contrast, other researchers found no significant association of triple negative phenotype with shorter local relapse-free survival (LRFS) [15,20]. Additionally, Haffty's observation revealed that the triple negative cohort had a predilection for relapse in regional nodes with marginal significance (*P *= 0.05) [15], which partly supported our findings.

Although Dent and colleagues observed a prominent increase in the rate of visceral metastasis for triple negative patients when compared with that for non-triple negative patients [21], it still remains unclear whether triple negative patients have a greater risk of visceral metastasis than ERBB2+ patients. We found that the risk of developing a visceral metastasis as the site of the first recurrence was significantly higher in women with triple negative and ERBB2+ breast cancers than in women with HR+/ERBB2- tumors. Besides, we also documented that the peak in the hazard rate for visceral metastasis was later for triple negative patients than ERBB2+ patients. These findings suggest that the pattern of visceral metastasis for the triple negative cases may be intermediate between that for HR+/ERBB2- and ERBB2+ groups in Chinese breast cancer patients.

Hormone receptor status has been accepted as sufficiently established to predict the risk of bone metastasis in breast cancer [22,23]. With the considerable understanding of intrinsic molecular subtypes, it is far from satisfactory to determine the clinical outcome exclusively by HR status. It has been reported that there was no difference in the rate of bone metastasis between triple negative and non triple negative groups [13,21]. Unfortunately, the previous studies overlooked the respective contribution of HR and ERBB2 status to the development of bone spread. In the present analysis, the triple negative phenotype brought about a dramatic increase in the hazard of developing bone metastasis with statistical significance compared with ERBB2+ subtype (*P *< 0.05). Furthermore, a similar trend was also observed between triple negative and HR+/ERBB2- groups with borderline significance (*P *= 0.058). In this sense, those with ERBB2+ tumors metastasized less often to bone than ERBB2- breast cancers, which was relatively consistent with Kallioniemi's findings [24]. Taken together, all of these data inferred that the ERBB2 status, besides HR status, should be taken into account when talking about the risk of bone metastasis, as exemplified by triple negativity.

This study has some potential and inevitable limitations due to its retrospective nature. Recurrences might be somewhat underreported or misinformed for a substantial portion of the patients in this database; nonetheless, underreporting or misinformation of recurrences would have not varied by clinicopathological parameters [25]. We did not evaluate the effect of treatment on survival in the present study, but all the hazard ratios were adjusted for treatment administered [11]. Furthermore, the limited sample size and the lack of more detailed molecular profiling are also potential weaknesses.

## Conclusion

In conclusion, our data show that preferential relapse sites for triple negative breast cancers may be quite distinct from that for HR+/ERBB2- and ERBB2+ counterparts in Chinese breast cancer patients. It indicates that different organotropism may be due to the different intrinsic subtypes. Therefore, a better knowledge of organotropism in different intrinsic subtypes is warranted for efficacious systemic treatment to decrease and/or delay the recurrence hazard.

## Competing interests

The authors declare that they have no competing interests.

## Authors' contributions

JL is responsible for editorial correspondence and has contributed to the conception and design of the study, the analysis and interpretation of data, the revision of the article as well as final approval of the version to be submitted. YL participated in the design of the study, performed the statistical analysis, interpretation of data, drafted and revised the article. WY participated in the design of the study, performed the statistical analysis, interpretation of data, and helped to draft and revise the article. TY participated in the design of the study and helped to revise the article. LZ participated in the design of the study and helped to revise the article. GD participated in the design of the study and helped to revise the article. JW conceived of the study, participated in its design and helped to revise the article. ZZS conceived of the study, and participated in its design and coordination. ZMS conceived of the study, and participated in its design and coordination. All authors read and approved the final manuscript.

## Pre-publication history

The pre-publication history for this paper can be accessed here:

http://www.biomedcentral.com/1471-2407/9/342/prepub
